# Supplementary material for: Multimodal imaging and functional analysis of the chick NMDA retinal damage model
Source: PLoS One. 2021 Sep 7;16(9):e0257148. doi: 10.1371/journal.pone.0257148 (PMC8423281; doi:10.1371/journal.pone.0257148)
Supplement: S1 File — (ZIP) [file pone.0257148.s004.zip › SD-OCT/C200727/013/D15-PWk3/C200727-013_OS_R_14_0_REGAVG0000049/Manual_PreDefineThreeByFourGridRadialRetina_1.pdf]

Three By Four Grid Radial Retina Template Report

**Patient ID**	C200727	**Name**	See InVivoVue
**Exam Date**	See InVivoVue	**Exam Time**	See InVivoVue
**Eye**	OS	**Averaged**	N/A
**Depth (mm)**	1.65	**Depth Samples**	1024
**Pitch (um/pix)**	14.000 x 2333.333 x 1.608	**Refractive Index**	See InVivoVue
**Scan Type**	Radial 14.000mm x 14.000mm x 1.647mm , 1000 x 6 x 1024		

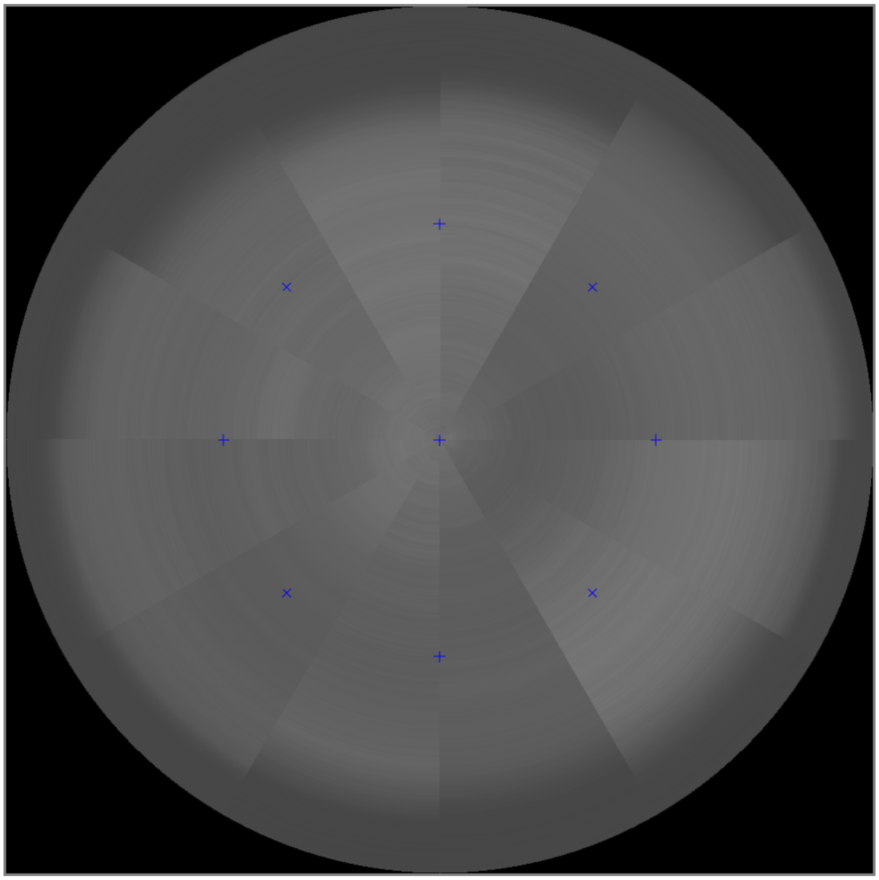

Thickness Metrics

Analysis	Average(mm)	Minimum(mm)	Maximum(mm)	STDEV(mm)
**IRNFL - RPE**	0.2562	0.2409	0.2765	0.0122
**IRNFL - ORNFL**	0.0295	0.0187	0.0474	0.0087
**ORNFL - OPL**	0.1503	0.1348	0.1741	0.0141
**OPL - ETPRS**	0.0567	0.0512	0.0612	0.0030
